# Enhancing soil health, microbial count, and hydrophilic methomyl and hydrophobic lambda-cyhalothrin remediation with biochar and nano-biochar

**DOI:** 10.1038/s41598-024-70515-2

**Published:** 2024-08-22

**Authors:** Kanchana Chandi, Patchimaporn Udomkun, Thirasant Boonupara, Puangrat Kaewlom

**Affiliations:** 1 Department of Environmental Engineering, Faculty of Engineering, Chiang Mai University, Chiang Mai, 50200 Thailand; 2 Office of Research Administration, Chiang Mai University, Chiang Mai, 50200 Thailand

**Keywords:** Soil amendment technologies, Sustainable agriculture, Organic carbon, Nano-structured material, Pesticide mitigation, Polar and non-polar pesticides, Environmental remediation, Biogeochemistry, Environmental sciences

## Abstract

Pesticide contamination and soil degradation present significant challenges in agricultural ecosystems, driving extensive exploration of biochar (BC) and nano-biochar (NBC) as potential solutions. This study examines their effects on soil properties, microbial communities, and the fate of two key pesticides: the hydrophilic methomyl (MET) and the hydrophobic lambda-cyhalothrin (LCT), at different concentrations (1%, 3%, and 5% w w^−1^) in agricultural soil. Through a carefully designed seven-week black bean pot experiment, the results indicated that the addition of BC/NBC significantly influenced soil dynamics. Soil pH and moisture content (MC) notably increased, accompanied by a general rise in soil organic carbon (SOC) content. However, in BC5/NBC5 treatments, SOC declined after the 2nd or 3rd week. Microbial populations, including total plate count (TPC), phosphate-solubilizing bacteria (PSB), and nitrogen-fixing bacteria (NFB), showed dynamic responses to BC/NBC applications. BC1/NBC1 and BC3/NBC3 applications led to a significant increase in microbial populations, whereas BC5/NBC5 treatments experienced a decline after the initial surge. Furthermore, the removal efficiency of both MET and LCT increased with higher BC/NBC concentrations, with NBC demonstrating greater efficacy than BC. Degradation kinetics, modeled by a first-order equation, revealed that MET degraded faster than LCT. These findings underscore the profound impact of BC/NBC on pesticide dynamics and microbial communities, highlighting their potential to transform sustainable agricultural practices.

## Introduction

The agricultural sector is crucial to global well-being, utilizing various pesticides to meet the demands of a growing population and enhance crop yields^1–3^. Over time, pesticide use has significantly increased, particularly in developing nations. Our group has reviewed the concentrations of pesticides reported in different countries, as detailed in Boonupara et al.^3^. Despite their essential role in food security, there is a growing international consensus to reduce pesticide usage due to their well-documented negative impacts on the environment and human health. Alarmingly, up to 80% of applied pesticides enter the soil, water, and air without reaching the intended target pests, leading to numerous adverse effects^4,5^.

Methomyl (MET), an oxime pesticide in the carbamate class, is widely used to control various pest stages, but its high-water solubility prevents it from being retained in the soil^6^. Its half-life ranges from 3 to 50 days in soil, 6–262 days in water, and 160–224 days in the air^7,8^. A survey in France reported that 4.2% of the population was directly or indirectly poisoned by methomyl between 2012 and 2016^9^. Due to its high residual toxicity towards mammals, birds, and the environment, methomyl has been banned in many European countries^9^, though it remains legal in Thailand. Conversely, lambda-cyhalothrin (LCT), a type II pyrethroid insecticide, is used to control various pests in homes and agricultural fields^10^. Its half-life in water and soil is approximately 30 days when exposed to sunlight^11^. Its high efficiency, easy biodegradation, and low toxicity make it more commonly used than organochlorines and organophosphates^12^. The United States, China, and India are the primary consumers and producers of lambda-cyhalothrin, while Thailand, Australia, Japan, Germany, Russia, and other countries are becoming significant consumers due to increased cultivated land area^11^. However, residues of this pesticide can persist in fields long after application, leading to toxicity issues in various organs of non-target organisms, with oxidative stress being the main cause^10,13^.

Given these challenges, it is imperative to reduce residual pesticide concentrations in the soil using effective remediation techniques. Various methods, such as soil washing, soil flushing, soil vapor extraction, and bioremediation, have been proposed for cleaning up contaminated soil. However, these methods often face significant limitations, including high maintenance costs, fertility loss, nutrient leaching, and soil erosion, or they may introduce new issues post-application^14,15^. The need for an environmentally friendly and sustainable approach to address soil pesticide contamination is evident, particularly given the lack of fully tested remediation technologies^16^. Consequently, the in-situ application of amendments to contaminated soil emerges as a straightforward, cost-effective, and energy-efficient option for remediation—a more sustainable alternative to pricier and hazardous methods^17^.

Biochar (BC), a carbon-rich byproduct from biomass pyrolysis under limited oxygen supply and moderate temperature conditions (< 700 °C), offers a high specific surface area, considerable porosity, and robust adsorption capacity, all at a relatively low cost^14^. BC can effectively reduce greenhouse gas emissions by sequestering carbon^18,19^. When added to soil, BC provides essential macro and micronutrients, acting as a cost-effective, slow-release fertilizer^20,21^. Numerous studies have documented the positive impact of BC on plant growth and yield, attributing these benefits to improvements in soil properties such as fertility, porosity, water-holding capacity, cation exchange capacity, and bulk density^22–29^. Moreover, BC has gained increased attention for its remarkable ability to decrease the bioavailability of pesticides^16,30–32^. However, the effectiveness of BC in remediating or immobilizing pesticides in soil depends on various factors, including the types of pesticides and soil, BC dosage and application methods, solution pH, and prevailing environmental conditions^19^.

With advancements in nanotechnology, researchers have explored the production of nano-biochar (NBC) for sustainable soil and agricultural applications^33^. NBC, derived from the pyrolysis of BC, results in micro-sized biochar with dimensions ranging from below a micrometer (µm) to nanometers (nm). The distinctions between bulk BC and NBC are characterized by structural variances and diverse physicochemical attributes. Unlike bulk BC, NBC exhibits a significantly smaller size, typically less than 100 nm, a higher surface area-to-volume ratio, a smaller hydrodynamic radius, a more negative zeta potential, and an increased presence of oxygen-containing functional groups^34–36^. These characteristics are influenced by factors such as feedstock material, production method, pyrolysis temperature, and other pre- or post-treatment methods^37^. The growing interest in NBC has led to versatile applications, particularly in agriculture, as demonstrated by studies conducted by Dong et al.^38^, Liu et al.^39^, Zhang et al.^40^, Rashid et al.^41^, and Chen et al.^42^.

Soil, a complex entity with multiple components, functions as an open biochemical system where microorganisms play a crucial role in maintaining ecosystem health and function. Changes in the microbial community can have profound effects on the entire ecosystem. To comprehensively assess the impact of BC and NBC on soil properties and microorganism abundance in pesticide-contaminated soil, we conducted a pot experiment using different concentrations of BC and NBC (1%, 3%, and 5% w w^−1^). This study takes a novel approach by specifically examining the hydrophilic and hydrophobic characteristics of selected pesticides—MET and LCT—in relation to their remediation using BC and NBC.

Unlike previous studies that have generally focused on the overall effectiveness of BC and NBC in improving soil properties and reducing pesticide bioavailability, this research provides a direct comparison of BC and NBC in removing pesticides with differing water affinities. By selecting MET and LCT, this study allows for a detailed exploration of the distinct mechanisms through which BC and NBC interact with hydrophilic versus hydrophobic contaminants. This research significantly enhances our understanding of the versatility and effectiveness of BC and NBC in soil remediation by demonstrating how their adsorption capacities and interactions with soil properties and microbial communities vary based on the water solubility of the pesticides. Previous studies by Liu et al.^39^ showed that NBC can markedly reduce the availability of cadmium (Cd) in soil, thereby enhancing the stability of Cd ions within the soil matrix. Concurrently, the introduction of NBC resulted in a significant increase in microbial biomass, as well as greater abundance and diversity of microorganisms. Rashid et al.^41^ found that the effects of NBC on soil microbial biomass, nutrient availability, and their uptake by corn crops are highly dependent on both concentration and nutrient levels. However, these studies did not address the comparative efficacy of BC and NBC in pesticide remediation. Therefore, this research fills that gap by offering valuable insights into the specific interactions of BC and NBC with pesticides of varying water solubility.

## Materials and methods

### Chemicals

MET (Lannate^®^ 98 SP, Beetle Industry Ltd., Lima, Peru) and LCT (2.5% w v^−1^ EC, Wanshope, Hitech Group Chemical Supply Co., Ltd., Mueang, Samutprakarn), locally acquired in the Mueang district of Chiang Mai, Thailand, were employed in the experiments. The physical and chemical properties of MET and LCT are summarized in Table 1. Chemicals and solvents for extraction and laboratory analysis were procured from RCI Labscan (Bangkok, Thailand). The quick, easy, cheap, effective, rugged, and safe (QuEChERS) extraction kit (Agilent Technologies, CA, USA), inclusive of magnesium sulfate (MgSO_4_), sodium chloride (NaCl), sodium citrate, and disodium citrate sesquihydrate (C_12_H_18_Na_4_O_17_), along with 2 mL of QuEChERS dispersive solid-phase extraction (SPE) containing primary secondary amine (PSA), octadecylsilane end-capped, and magnesium sulfate, was utilized for purification purposes. Standard references of MET and LCT, with a purity exceeding 99% for LC–MS/MS analysis, were sourced from CPAchem Ltd. (Bogomilovo, Bulgaria).

**Table 1:** Physical and chemical properties of methomyl (MET) and lambda-cyhalothrin (LCT).

Properties	Methomyl (MET)	Lambda-cyhalothrin (LCT)	References
Molecular formula	C_5_H_10_N_2_O_2_S	C_23_H_19_ClF_3_NO_3_	^43^
Molecular weight (g mol^−1^)	162.21	449.85	^43^
Molecular structure			^43^
Water affinity	Hydrophilic	Hydrophobic	^44,45^
Water solubility (g L^−1^)	57.9 at 25 °C	pH 5: 4 × 10^−6^ pH 6.5: 5 × 10^−6^ pH 9.2: 4 × 10^−6^ at 20 °C	^44,45^
Octanol–water partition coefficient (log K_ow_)	0.6	7.0	^44,45^
Soil adsorption coefficient (K_oc_, cm^3^ g^−1^)	72	247,000–330,000	^46^
Half-life in soil (days)	3–50	22–82 at 20 °C 8.5–10.8 at 28 °C	^47–49^
Half-life in water (days)	6–262	21.9 (aerobic)	^46,47^
Half-life in air (days)	160–224		^47^
Photolysis half-life (days)	Water at pH 5 and 25 °C–2–3, with acetonitrile the principal product	Water at pH 5 and 25 °C–24.5 Soil–53.7	^44–46^
Hydrolysis half-life (days)	pH 5–Stable for 30 days pH 7–Stable for 30 days pH 9–50% loss in 30 days, with conversion to S-methyl N-hydroxythioacetimidate	pH 5–Stable pH 7–Stable pH 9–8.66	^44–46^
Acceptable daily intake (ADI, mg kg^−1^ bw day^−1^)	0.0025	0.0025	^50^
Acute reference dose (ARfD, mg kg^−1^ bw day^−1^)	0.0025	0.005	^50^

### Biochar and nano-biochar preparation

To produce BC and NBC, bamboo sourced from an agricultural field in the San Pa Tong district of Chiang Mai, Thailand, was used. The bamboo was initially cleaned with distilled water to remove accumulated dirt and then heated at 550 °C for 1 h to produce BC. The resulting BC underwent several processing steps to obtain NBC. First, the BC was frozen at − 80 °C for 24 h to facilitate further processing. It was then ground using a crushing machine (GM/EP-100×60X, Guang Ming, China) and further reduced in size using a hammer mill (GM/PC400×200A, Guang Ming, China). The ground material was sieved through a No. 4 sieve to obtain BC with a particle size range of 2–3 mm. Finer particles were separated using an ASTM standard test sieve No. 200. These fine particles were then processed with a high-energy planetary ball mill (Pulverisette 6, Fritsch, Germany) to produce NBC. The milling conditions were based on the method described by Naghdi et al.^51^, with modifications made to enhance particle size reduction. The final products were analyzed using scanning electron microscopy (SEM) and transmission electron microscopy (TEM) to verify that the particle size was within the nanometer range. Additionally, all BC and NBC samples underwent various physicochemical analyses, as detailed below.

Chemical composition analysis was performed using an organic elemental analyzer (Flash 2000, Thermo Scientific, MA, USA). Porosity was assessed through nitrogen (N_2_) sorption–desorption isotherms at 77 K, utilizing a Micromeritics instrument (ASAP2460, Micromeritics Instrument Co., Ltd., Norcross, USA). The point of zero charge (pH_PZC_) was determined using a mass titration method, following the methodology described by Abewaa et al.^52^. Erlenmeyer flasks were filled with 50 mL of 0.1 M NaCl solution, and the pH was adjusted to range from 1 to 12 using 0.1 M hydrochloric acid (HCl) or 0.1 M sodium hydroxide (NaOH). Subsequently, 1 g of BC or NBC sample was added to each flask, and the mixtures were shaken at 125 rpm for 48 h. A digital pH meter was used to measure the final pH of each solution. The pH_PZC_ was determined by plotting the initial pH against the difference between the initial and final pH values. The pH_PZC_ was identified as the point where the initial pH equals the final pH.

Particle size distribution was assessed using a laser particle size distribution analyzer (CILAS 1190, CILAS, Les Ulis, France). Functional groups were analyzed using a Fourier-Transform Infrared (FTIR) spectrometer (Tensor27, Bruker, Ettlingen, Germany). The sample powder was diluted in 1% (w v^−1^) potassium bromide (KBr), and the FTIR spectrum, covering the range of 400–4000 cm^−1^, was measured with a resolution of 4 cm^−1^. The surface morphology of both BC and NBC samples was analyzed using SEM (JEM2100, Merlin compact, Carl Zeiss, USA) with an applied high tension of 5.0 kV, revealing detailed structural characteristics. TEM (JEM1400, JEOL, Japan was employed at an accelerating voltage of 80 kV, providing high-resolution imaging and intricate structural details. For sample preparation, 0.2 g of NBC was ultrasonically dispersed in ethanol. A droplet of the resulting suspension was then placed onto a copper grid and coated with a thin carbon film.

### Soil and pesticide-contaminated soils preparation

Collected from the 0–20 cm layer in the San Pa Tong district of Chiang Mai, Thailand, the black soil originates from an area consistently dedicated to the cultivation of black beans (*Phaseolus vulgaris*). The soil samples were air-dried, sieved to < 2 mm, and homogenized before use. To introduce MET and LCT into the soil, a solution of MET and LCT in methanol was prepared and added to the soil. The treated soil was placed in a fume cupboard until the methanol completely evaporated. After the evaporation of methanol, the treated soil was gradually mixed with uncontaminated soil and homogenized until reaching target concentrations of 2.0 mg kg^−1^ dry weight for MET and 2.5 mg kg^−1^ dry weight for LCT. This stepwise mixing process facilitated the preparation of 20 kg of each pesticide-contaminated soil, with an equal amount of uncontaminated soil set aside as a control. After mixing, three random samples were selected to determine MET and LCT concentrations, revealing differences in results of less than 5%. The recovery rates of MET and LCT in the soil ranged from 97.6 to 101.2%.

### Pot experiment and sample collection

The experiments were conducted in polyvinyl chloride (PVC) plastic pots with dimensions of 8 cm in bottom diameter, 15 cm in top diameter, and 11 cm in height, each containing 1 kg of soil. The MET-contaminated and LCT-contaminated soils were divided into three portions. The first portion served as the control. The second portion was uniformly mixed with BC at three different levels (1%, 3%, and 5% w w^−1^), denoted as MET-BC and LCT-BC, respectively. The third portion was mixed with NBC at three different levels (1%, 3%, and 5% w w^−1^), referred to as MET-NBC and LCT-NBC, respectively.

Seeds of a local black bean cultivar were obtained from a market in the Mueang district of Chiang Mai, Thailand. To ensure sterility, the seeds were surface-sterilized using a 2% (v v^−1^) sodium hypochlorite solution, followed by thorough washing and soaking in deionized water at room temperature for 1 h. The germination process took place in seed-starter trays, and only healthy, germinated seeds were selected for transplantation into the pots, with each pot accommodating five seedlings. All pots were placed in a greenhouse without additional fertilization, maintaining daytime temperatures between 28 and 30 °C, nighttime temperatures between 20 and 25 °C, and humidity levels between 50–75%. Daily watering with sterile deionized water was carefully administered to maintain soil moisture levels at 60–70% (w w^−1^).

Soil samples, approximately 100 g each, were collected weekly for seven weeks. Each meticulously collected soil sample was sealed in a reclosable zip-lock bag and accurately labeled for identification. The samples were promptly transported to the laboratory and stored at room temperature for less than seven days before undergoing subsequent analytical procedures.

### Sample analyses

#### Examination of soil properties

The soil samples underwent a series of preparatory steps, including air-drying, fine grinding, and sieving through a 0.15 mm mesh to remove larger debris or aggregates. Soil organic carbon (SOC) was determined using the Walkley–Black method^53^. Soil moisture content (MC) was measured by drying the samples in a hot air oven (model LDO-060E, DAIHAN LABTECH Co., Ltd., Gyeonggi, Republic of Korea) at 105 °C until a constant weight was achieved, and the dry weight of the samples was recorded. For pH assessment, a soil and ultrapure water suspension (1:2.5 w v^−1^) was prepared, and the pH was measured using a glass pH electrode (EZ-9909SP, Jinan Runjie Electronic Technology Co., Ltd., Shandong, China). All analyses were conducted in triplicate.

#### Qualitative analysis of microbial ability

To prepare the nutrient agar medium for total plate count (TPC), the suspension was heated until the medium was completely dissolved, and the pH was adjusted to 7.4. Pikovskaya’s medium, used for phosphate-solubilizing bacteria (PSB), was adjusted to a pH of 7.0. For identifying nitrogen-fixing bacteria (NFB), a modified nitrogen-free medium based on Jensen’s method^54^ was utilized, with the medium’s pH set to 7.6. All media were autoclaved (model LAC-5060SD, DAIHAN LABTECH Co., Ltd., Gyeonggi, Republic of Korea) at 121 °C for 15 min.

For analysis, each soil sample (1 g) was dispersed in 9 mL of autoclaved distilled water and filtered through 125 mm Whatman^®^ qualitative filter paper (Whatman, Merck Ltd., Bangkok, Thailand). Subsequently, 1 mL of the filtered solution was transferred to 9 mL of sterile distilled water to create a 10^−2^ dilution. Serial dilutions (10^−3^, 10^−4^, 10^−5^, 10^−6^, and 10^−7^) were then prepared for each soil sample. From the serially diluted (10^−4^, 10^−5^, and 10^−6^) soil samples, 0.1 mL volumes were plated on each medium and incubated (model Accuplus i250, Entech Industrial Solution Co., Ltd., Rayong, Thailand) at 30 °C for 24 h for TPC, 35 °C for 24 h for PSB, and 30 °C for 4 days for NFB.

#### Quantification of methomyl and lambda-cyhalothrin

The extraction of soil samples followed the QuEChERS method as detailed below: A precise 10 g of soil was weighed and placed in a 50 mL centrifuge tube. Then, 10 mL of water was added, and the mixture was allowed to stand for 30 min. Next, 10 mL of acetonitrile was added, and the tube was sealed and shaken vigorously for 1 min. A buffer-salt mixture (4 g MgSO_4_, 1 g NaCl, and 0.5 g C_12_H_18_Na_4_O_17_) was introduced to aid in phase separation and pesticide partitioning. After resealing and shaking for an additional minute, the tube was centrifuged (VARISPIN 4, NOVAPRO Co., Ltd., Seoul, Republic of Korea) at 4000 rpm for 5 min. One milliliter of the acetonitrile phase was transferred to a separate tube containing 150 mg of MgSO_4_ and 25 mg of PSA. After sealing and shaking for 30 s, the tube was centrifuged using a microliter and hematocrit centrifuge (NF 480, NÜVE SANAYİ MALZEMELERİ İMALAT VE TİCARET A.Ş, Ankara, Turkey) at 4000 rpm for 5 min. The residue was reconstituted with 0.5 mL of acetonitrile. Each final extract was filtered through a 0.2 µm membrane filter into a 2 mL amber glass vial and stored at − 20 °C until LC–MS/MS analysis.

The LC–MS/MS system included a 1290 vialsampler (G7129B, Agilent, California, USA), a 1290 high-speed pump (G7120A, Agilent, California, USA), and a 1290 MCT detector (G7166B, Agilent, California, USA). Separation was performed on a Phenomenex Luna C18(2) column (150 × 2.00 mm ID, particle size 5 µm). The eluents consisted of water containing 0.1% formic acid and 5 mM ammonium acetate (CH_3_CO_2_NH_4_) (A) and methanol containing 0.1% formic acid and 5 mM CH_3_CO_2_NH_4_ (B). The flow rate was set at 0.3 mL min^−1^. The autosampler and column temperatures were maintained at 4 °C and 40 °C, respectively, with a 2 µL injection volume. Detection was achieved using Agilent Jet Stream electrospray ionization (ESI) in positive mode, with a capillary voltage ranging from 3200 to 3800 V, nebulizer pressure at 45 psi, sheath gas temperature at 400 °C with a sheath gas flow of 12 L min^−1^, and gas temperature at 300 °C with a gas flow of 3 L min^−1^. Collision gas pressure and tube lens offset voltages were optimized using the automated optimization procedure. The mass spectrometry scanning method employed dynamic multiple reaction monitoring (MRM), and the analysis was performed in triplicate.

### Data analysis

In this investigation, the degradation rate constant (k) and half-lives (t_1/2_) were computed using the methodology outlined by Sun et al.^55^ based on the first-order rate equation Eq. (1):

1 $${C}_{t}={C}_{0}^{-kt}$$

where C_t_ represents the concentration of pesticide residue at time t, C_0_ is the initial concentration after application, and k is the degradation rate constant (days⁻^1^). The t_1/2_ was then derived from the k value using Eq. (2):

2 $${t}_{1/2}=ln\left(\frac{2}{k}\right)$$

The soil properties and pesticide residue data were presented as mean ± standard deviation (SD). Statistical analysis was performed using the general linear model (GLM) program. To discern significant differences among treatment means at a 5% probability level, Fisher’s least significant difference (LSD) test was applied through the SAS program (version 9.4, SAS Institute, 2002). Linear and non-linear regression analyses were conducted using Microsoft Excel 2023.

## Results and discussion

### Comparison of characteristics between biochar and nano-biochar

**Fig. 1:**
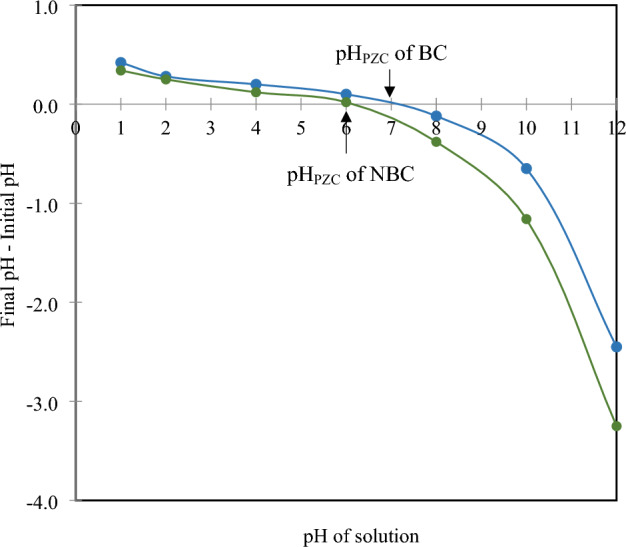
pH point of zero charge (pH_PZC_) of biochar (BC) and nano-biochar (NBC) at different pH values.

A comparative analysis of the elemental composition and essential physical properties of BC and NBC is outlined in Table 2. Carbon is the predominant element in both materials, constituting 74% by weight, followed by oxygen at 23.4%, hydrogen at 1.8%, and nitrogen at 0.7%. The pH_PZC_ was used to assess the surface charge characteristics of BC and NBC, as shown in Fig. 1. The pH_PZC_ of BC was found to be 7, while that of NBC was 6, indicating distinct differences in their surface chemistry and environmental interactions. These pH_PZC_ values suggest that NBC likely possesses a higher surface area or a greater number of functional groups compared to BC, which influences its adsorption capacities and interactions with pollutants and nutrients^56^. The lower pH_PZC_ of NBC implies that it may be more effective in adsorbing anions under acidic conditions and cations under basic conditions, enhancing its utility in environmental remediation where precise adsorption properties are critical^36^.

**Table 2:** Physicochemical properties of biochar (BC) and nano-biochar (NBC) derived from bamboo pyrolyzed at 500 °C.

Parameters	Biochar (BC)	Nano-biochar (NBC)
Carbon (C, % w w^−1^)	74.46 ± 0.24	74.12 ± 0.22
Hydrogen (H, % w w^−1^)	1.88 ± 0.02	1.83 ± 0.02
Oxygen (O, % w w^−1^)	23.21 ± 0.25	23.36 ± 0.26
Nitrogen (N, % w w^−1^)	0.46^b^ ± 0.02	0.69^a^ ± 0.01
O/C ratio ( −)	0.31 ± 0.01	0.32 ± 0.04
H/C ratio ( −)	0.03 ± 0.00	0.02 ± 0.01
C/H ratio ( −)	39.60 ± 0.17	40.50 ± 0.07
C/N ratio ( −)	161.87^a^ ± 0.31	107.42^b^ ± 0.13
Surface area (m^2^ g^−1^)	144.52^b^ ± 15.20	756.22^a^ ± 30.25
Total pore volume (× 10^−6^ m^3^ g^−1^)	0.18^b^ ± 0.01	0.32^a^ ± 0.28
Average pore diameter (nm)	8.15^a^ ± 0.14	1.65^b^ ± 0.22

All values show the mean ± standard deviation.

^a,b^signifies significant differences according to the LSD test (p < 0.05).

**Fig. 2:**
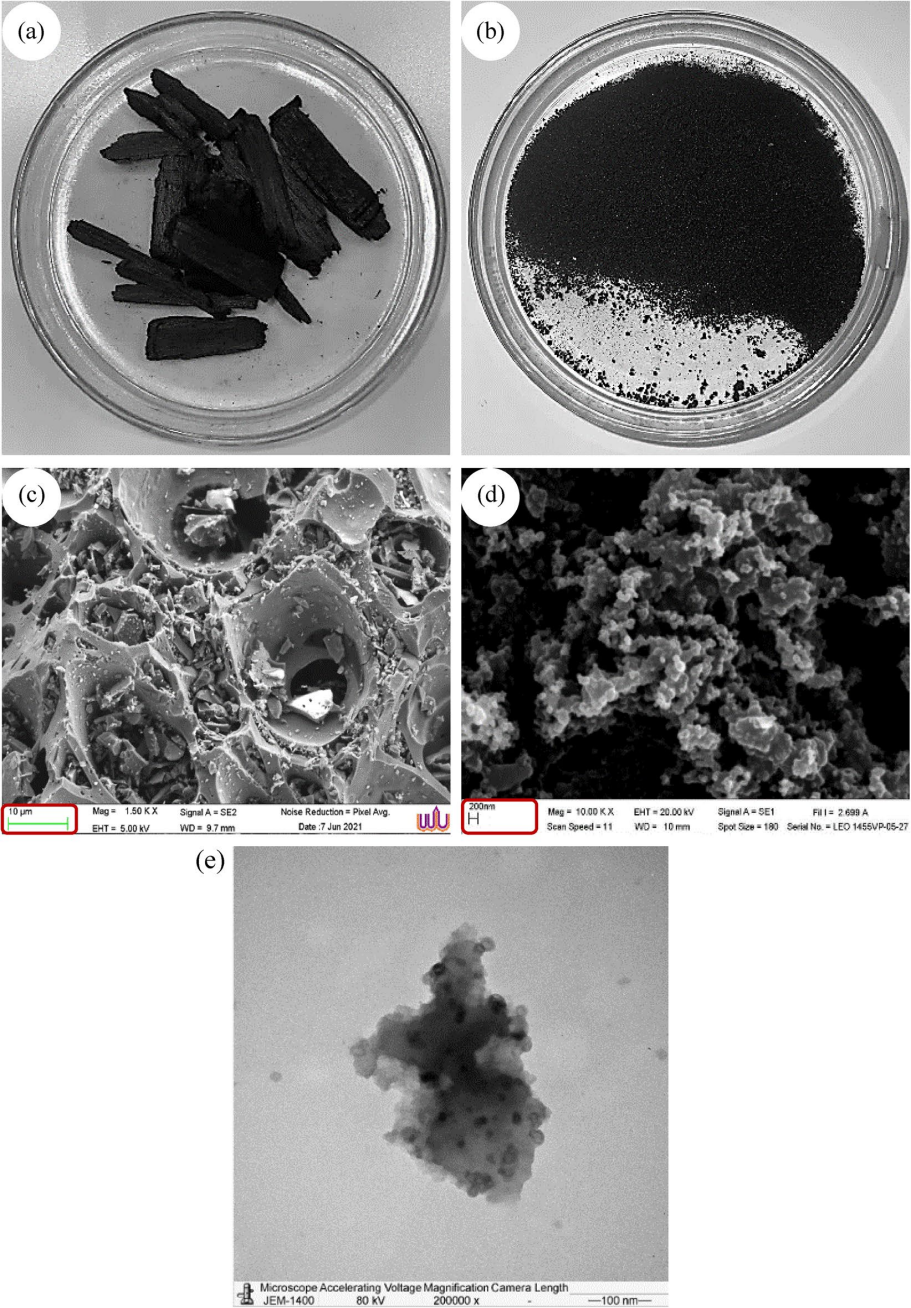
Visual appearance of (**a**) biochar (BC) and (**b**) nano-biochar (NBC). Scanning electron microscopy (SEM) images: (**c**) BC (×1500) and (**d**) NBC (×10,000), illustrating the micrometer-scale structure of BC and the nanometer-scale structure of NBC. Transmission electron microscopy (TEM) image: (**e**) NBC (×200,000), showing the detailed nanometer-scale structure of NBC.

Moreover, BC exhibits a surface area of 144.5 m^2^ g^−1^ and an average pore volume of 0.15 × 10^–6^ m^3^ g^-1^, whereas NBC demonstrates a significantly larger surface area of 756.2 m^2^ g^−1^ and a pore volume of 0.3 × 10^−6^ m^3^ g^−1^. This results in a much higher average surface area-to-volume ratio for NBC (2363.2 × 10^6^ m^−1^) compared to BC (802.9 × 10^6^ m^−1^). The pore diameter is 8.2 nm for BC and 1.7 nm for NBC. SEM and TEM analyses of both BC and NBC revealed notable differences in surface morphology (Fig. 2). These analyses confirmed that NBC particles were indeed within the nanometer range, validating the effectiveness of the processing steps in achieving nano-size particles. Likewise, NBC exhibited a significantly more porous structure and smaller particle size compared to BC, indicating an increased surface area and enhanced adsorption capacity.

**Fig. 3:**
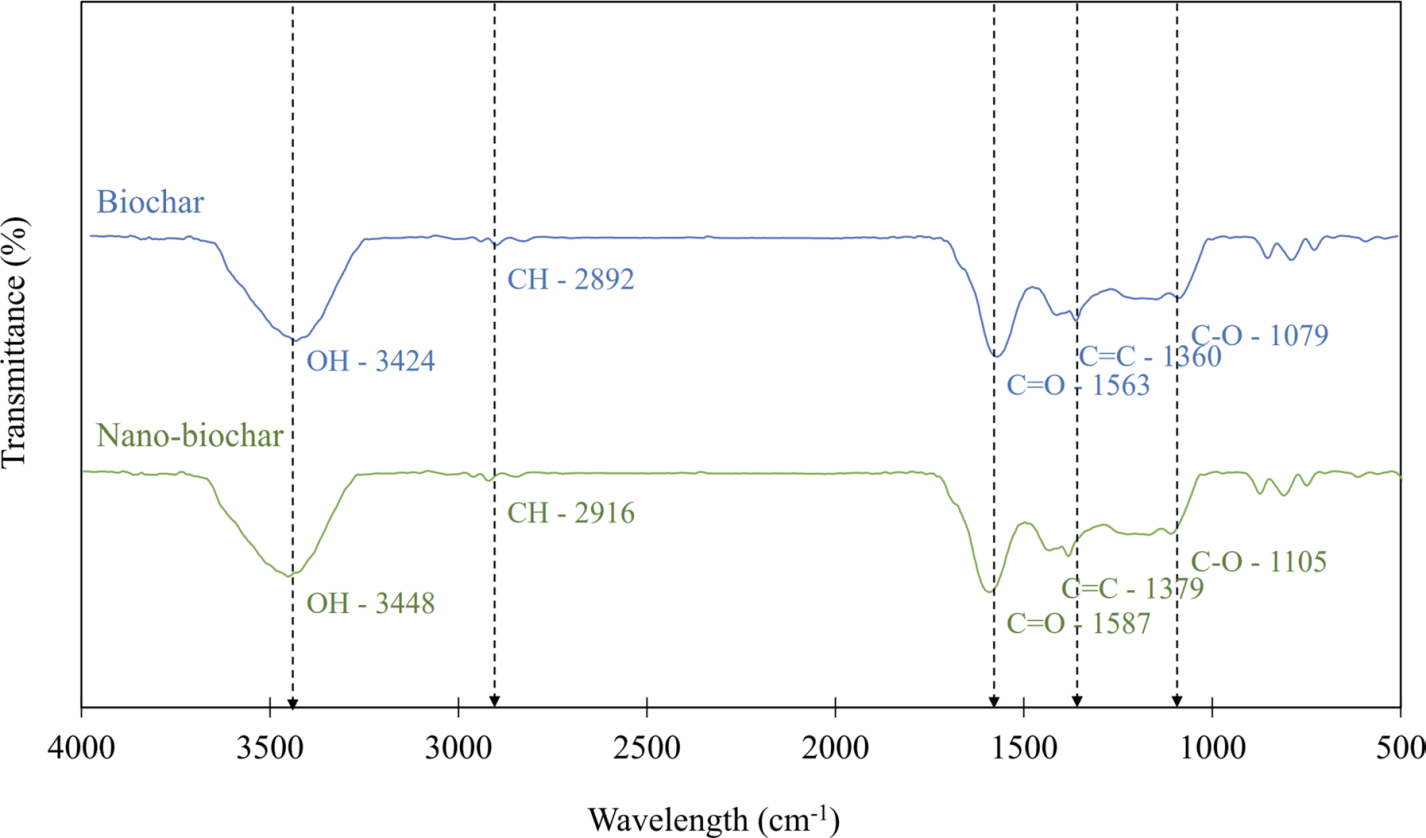
FTIR spectra of raw bamboo, biochar (BC), and nano-biochar (NBC).

BC is known for its carbonaceous composition, characterized by the presence of various oxygen-containing functional groups, such as carboxylic (–COOH) and hydroxyl (–OH) groups, distributed across its surface^57^. These functional groups, combined with its porous structure, make BC a versatile material with diverse applications. A significant deviation in the FTIR spectra between BC and NBC was observed, indicating biological alterations in their functional groups. The band intensity between 726–871 cm^−1^ (Fig. 3) suggests the presence of aromatic C–H bonds, corresponding to the aromatic benzene rings on the BC/NBC surfaces^58^. Prominent bands in the 1000–1500 cm^−1^ range for both BC and NBC indicate an abundance of –OH and –COOH groups, which are prone to complexation with metal ions^59^. Peaks observed at 1360–1379 cm^−1^ correspond to the stretching vibrations of double C = C bonds in aromatic rings. Another notable feature is the carbonyl group (C = O), found in organic molecules such as ketones, carboxylic acids, and esters, identified at 1563–1587 cm^−1^. These functional groups also indicate H-bonding interactions^60^. Peaks in the 2892–2916 cm^−1^ range are attributed to –CH stretching, indicating the presence of alkyl groups. Furthermore, peaks in the 3424–3448 cm^−1^ range indicate the presence of surface –OH groups on the BC/NBC surfaces, representing substantial H-bonding interactions^61^. Although Aziz et al.^62^ emphasized that NBC and BC share similar peaks, they noted that NBC exhibits more intense peaks and a greater variety of functional groups, including newly emerging peaks such as primary alcohol stretching and aliphatic chains, compared to BC. However, this observation may not necessarily apply to BC derived from bamboo, as examined in this study.

### Influence of biochar and nano-biochar on soil properties

**Fig. 4:**
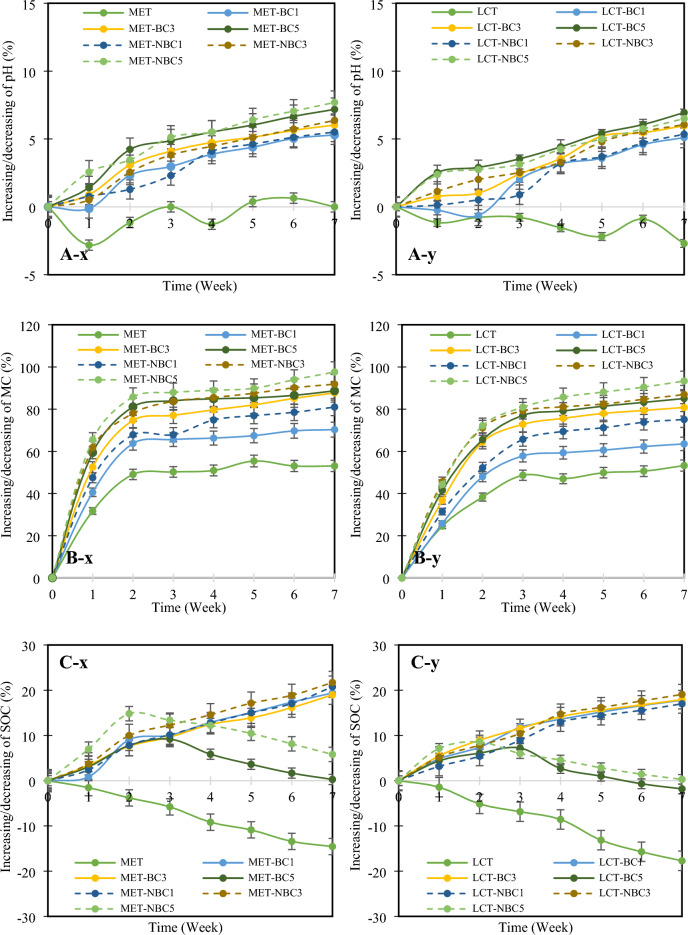
Variations of pH (**A**), moisture content (MC) (**B**), and soil organic carbon (SOC) (**C**) upon incorporating biochar (BC) and nano-biochar (NBC) at 1%, 3%, and 5% (w w^−1^) in soil contaminated with methomyl (MET) (x) and lambda-cyhalothrin (LCT) (y).

The chemical characteristics, including pH, MC, and SOC, of soils contaminated with MET and LCT in response to the addition of BC and NBC are illustrated in Fig. 4. In the absence of BC/NBC, the pH of MET- and LCT-contaminated soils fluctuated and generally decreased over the 7 week period (Fig. 4A-x, A-y). However, the introduction of BC/NBC led to a significant increase (p < 0.05) in soil pH compared to soils without BC/NBC. The pH increase was more pronounced with higher BC/NBC levels. Specifically, at the 7th week, MET-NBC1% showed a 5.5% increase compared to the initial week, while MET-NBC5% exhibited a 7.7% rise. The pattern of pH change with BC/NBC application in hydrophilic MET and hydrophobic LCT-contaminated soils was almost identical.

The observed rise in pH following the addition of BC is likely attributed to the substantial ash content and base cations present in BC. The alkalinity inherent in BC, coupled with the subsequent release of key base cations, particularly Ca^2+^ and K^+^, facilitates the displacement of soil-exchangeable Al^3+^ and H^+^ ions. This displacement, occurring on the negatively charged sites of the soil, significantly elevates the soil pH^63^. Moreover, the negatively charged functional groups, such as phenolic, carboxylic, and hydroxyl groups, located on the surface of biochar, contribute to the pH increase by binding surplus H⁺ ions in the soil solution^64^. Another potential mechanism involves the decarboxylation of organic anions induced by the addition of BC. This process, initiated by increased microbial activity on organic anions, leads to the consumption of excess H⁺ ions from the soil solution and consequently raises the soil pH^65^.

In contrast, Khader et al.^66^ reported different results. They observed a decrease in soil pH during tomato cultivation in sandy loam soil when nano-amendments, such as NBC, green-NBC, and magnetic-NBC, were applied at levels of 3 and 6 mg kg^−1^. The observed pH reduction was attributed to the organic acids generated during the decomposition of organic matter within the soil and NBC. To reconcile these conflicting outcomes, Hossain et al.^26^ proposed that the impact of BC application on soil pH is contingent upon the initial soil properties (such as pH and texture) and the pH and alkalinity of the biochar itself.

Considering MC, it is evident that soil contaminated with MET and LCT exhibited an increase over the 7 week period (Figs. 4B-x, B-y). The dynamics of MC in soil contaminated with MET and LCT were quite similar, with both experiencing a 53.1% and 53.3% increase, respectively, from the initial stage to the 7th week. Additionally, the application of BC/NBC led to a noticeable rise in soil MC that corresponded to the level of BC/NBC added. The increase in MC with NBC application tended to be slightly higher than with BC application. By the 7th week, a significant increase (p < 0.05) in soil MC was observed in both MET-BC5 (88.7%) and MET-NBC5 (97.6%), as well as in LCT-BC5 (85.0%) and LCT-NBC5 (93.3%). Furthermore, the relationship between soil MC and time (t) is depicted in Table 3, showing a nonlinear correlation characterized by the natural logarithm, with notably high coefficients of determination (R^2^ > 0.8 for MET and > 0.9 for LCT). Regarding regression results, the rate of increase in soil MC was most pronounced in BC5/NBC5, followed by BC3/NBC3, BC1/NBC1, and soil without pesticides.

**Table 3:** Regression results for soil moisture content (MC) with the introduction of biochar (BC) and nano-biochar (NBC) at 1%, 3%, and 5% (w w^−1^) over time (t) in soil contaminated with methomyl (MET) and lambda-cyhalothrin (LCT).

Treatment	Equation	R^2^
Soil contaminated with MET		
MET	MC = 5.482ln(t) + 23.352	0.864
MET-BC1	MC = 6.944ln(t) + 24.685	0.846
MET-BC3	MC = 8.020ln(t) + 24.522	0.868
MET-BC5	MC = 8.348ln(t) + 25.323	0.813
MET-NBC1	MC = 7.598ln(t) + 26.097	0.820
MET-NBC3	MC = 8.842ln(t) + 26.363	0.837
MET-NBC5	MC = 9.006ln(t) + 26.700	0.817
Soil contaminated with LCT		
LCT	MC = 5.229ln(t) + 21.868	0.936
LCT-BC1	MC = 7.431ln(t) + 25.25	0.929
LCT-BC3	MC = 8.294ln(t) + 23.375	0.918
LCT-BC5	MC = 8.895ln(t) + 24.397	0.921
LCT-NBC1	MC = 8.467ln(t) + 24.176	0.957
LCT-NBC3	MC = 8.936ln(t) + 25.092	0.916
LCT-NBC5	MC = 9.242ln(t) + 23.318	0.929

These findings corroborate previous research indicating a significant increase in soil MC with the addition of BC^67,68^. This phenomenon is due to BC’s inherent ability to retain water within its internal pores, directly enhancing soil MC. Factors such as the total pore volume and hydrophilic functional groups on the BC surface may also contribute to this observed enhancement^68^. Additionally, incorporated BC particles can act as binding agents, promoting the formation of larger soil microaggregates, which improves pore connectivity and enhances soil structure, ultimately increasing water-holding capacity. The slightly higher soil MC with NBC application can be explained by Tan et al.^69^, who proposed that NBC, with its high total pore volume and small pores, enhances capillary forces, thereby increasing water-holding capacity. However, Chen et al.^70^ reported contrasting results, suggesting that the application of BC with particle sizes ranging from 40 to 200 nm reduced soil water-holding capacity. They explained that NBC decreased the air-entry value, facilitating easier water escape from soil pores and negatively affecting water retention. Furthermore, NBC particles exhibited strong water repellency, altering the distribution of water within the soil pore structure^70^. This discrepancy might be attributed to differences in the size of NBC utilized, as the NBC used in this study was larger. In this study, the non-linear relationship between soil MC and time, observed both with and without BC/NBC application, also suggests the involvement of various factors beyond those explicitly measured or mentioned. Factors such as soil properties (including texture and structure), microbial activity, root growth, and the decomposition of organic matter might play significant roles in shaping soil moisture dynamics over time.

The analysis of SOC content reveals a notable decline over time in soils where BC/NBC was not applied. Specifically, reductions of 14.6% and 17.7% were observed in soil contaminated with MET and LCT, respectively, compared to their initial levels (Fig. 4C-x, C-y). Conversely, the incorporation of BC/NBC into the soil exerted a positive and significant influence (p < 0.05) on SOC content throughout the cultivation period. Notably, BC1/NBC1 and BC3/NBC3 treatments exhibited a sustained increase in SOC content. However, BC5/NBC5 exhibited a contrasting trend, with SOC content decreasing after the 3rd week for soils treated with BC5 and after the 2nd week for soils treated with NBC5. An interesting observation arises when comparing soils contaminated with MET to those contaminated with LCT. SOC levels with BC/NBC application showed slight variation, with some soils exhibiting higher levels. Moreover, a linear correlation between SOC and cultivation time (R^2^ > 0.94) was found in soils treated with BC1/NBC1 and BC3/NBC3, as well as in soils without pesticides (Table 4). In contrast, a non-linear correlation was observed in soils treated with BC5/NBC5 contaminated with both MET and LCT (R^2^ > 0.8).

**Table 4:** Regression results for soil organic carbon (SOC) with the introduction of biochar (BC) and nano-biochar (NBC) at 1%, 3%, and 5% (w w^−1^) over time (t) in soil contaminated with methomyl (MET) and lambda-cyhalothrin (LCT).

Treatment	Equation	R^2^
Soil contaminated with MET		
MET	SOC = − 0.095(t) + 3.612	0.982
MET-BC1	SOC = 0.083(t) + 3.374	0.950
MET-BC3	SOC = 0.098(t) + 3.520	0.981
MET-BC5	SOC = − 0.022(t^2^) + 0.189(t) + 3.442	0.808
MET-NBC1	SOC = 0.097(t) + 3.409	0.981
MET-NBC3	SOC = 0.102(t) + 3.462	0.941
MET-NBC5	SOC = − 0.030(t^2^) + 0.286(t) + 3.235	0.845
Soil contaminated with LCT		
LCT	SOC = − 0.087(t) + 3.613	0.995
LCT-BC1	SOC = 0.085(t) + 3.422	0.941
LCT-BC3	SOC = 0.090(t) + 3.473	0.930
LCT-BC5	SOC = − 0.016(t^2^) + 0.121(t) + 3.509	0.867
LCT-NBC1	SOC = 0.088(t) + 3.412	0.968
LCT-NBC3	SOC = 0.093(t) + 3.437	0.961
LCT-NBC5	SOC = − 0.015(t^2^) + 0.113(t) + 3.504	0.830

The response of SOC to the addition of BC and NBC can be attributed to the recalcitrant nature of carbon present in BC^71^. BC, especially when prepared at higher temperatures (above 400–500 °C), contains highly stable forms of carbon due to aromatization and the formation of larger aromatic ring complexes, which resist both biotic and abiotic degradation^72^. Consequently, adding BC with stable carbon to soils increases SOC levels. Despite its recalcitrant nature, BC can influence carbon cycle dynamics through microbial activity, potentially leading to positive priming effects on native organic matter and enhancing the degradation of native SOC. The observed linear correlation between SOC and cultivation time in soils treated with BC1/NBC1 and BC3/NBC3 supports a consistent and predictable increase in SOC content over time. However, the reduction in SOC observed with BC5/NBC5 application may be attributed to the negative priming effect of BC, as described by Jien et al.^73^, Zhu et al.^74^, and Yang et al.^75^. These authors outline various mechanisms contributing to this negative effect, including substrate switching, substrate dilution, direct inhibition of microbial activity, and the adsorption of native SOC by BC. Changes in soil microbial community composition, such as an increase in the fungi-to-bacteria ratio, can further suppress native SOC degradation and lead to substantial negative priming effects^76^. The non-linear correlation detected in BC5/NBC5-treated soils contaminated with MET and LCT suggests a more complex relationship between SOC and cultivation time, where additional factors such as BC’s adsorption properties and changes in soil microbial communities may play a role. Nonetheless, it is important to recognize that the effects of BC/NBC may vary based on feedstock characteristics, pyrolysis conditions, soil types, and the duration of impact^74^.

### Influence of biochar and nano-biochar on soil microbial characteristics

**Fig. 5:**
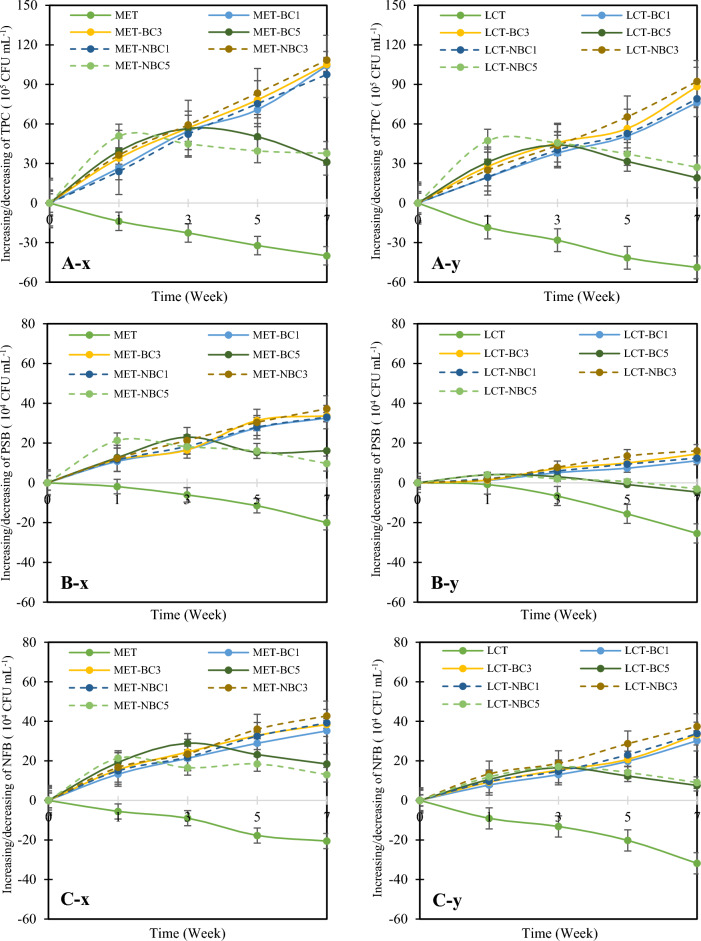
Changes in total plate count (TPC) (**A**), phosphate-solubilizing bacteria (PSB) (**B**), and nitrogen-fixing bacteria (NFB) (**C**) with the application of biochar (BC) and nano-biochar (NBC) at 1%, 3%, and 5% (w w^−1^), observed in soil contaminated with methomyl (x) and lambda-cyhalothrin (y).

The impact of BC/NBC on TPC, PSB, and NFB in soil contaminated with MET and LCT over a 7 week period is illustrated in Fig. 5. In soils contaminated with MET or LCT, TPC, PSB, and NFB exhibited a declining trend over time. However, a distinct pattern emerged with the application of BC/NBC. BC1/NBC1 and BC3/NBC3 significantly increased (p < 0.05) TPC, PSB, and NFB in both MET- and LCT-contaminated soils compared to soils without BC/NBC. Specifically, the number of TPC in MET-contaminated soil increased by over 97.5% with the application of BC1/NBC1 and BC3/NBC3 (Fig. 5A-x), while a substantial increase of more than 76.0% was observed in LCT-contaminated soil (Fig. 5A-y). Similarly, the population of PSB rose by 33.1% in MET-contaminated soils (Fig. 5B-x) and by 12.5% in LCT-contaminated soils (Fig. 5B-y). Additionally, NFB counts showed significant increases, with increments exceeding 35.2% in MET-contaminated soil (Fig. 5C-x) and reaching 30.2% in LCT-contaminated soil (Fig. 5C-y).

In contrast, in BC5-treated soil, decreases in TPC, PSB, and NFB were observed after the 3rd week, while NBC5 application resulted in declines in most microbial counts after the 2nd week. For instance, in MET-contaminated soil, TPC decreased from 59.2% in the 3rd week to 31.1% in the 7th week with BC5 application, and from 50.9% in the 2nd week to 37.7% in the 7th week with NBC5 addition. This trend was consistent across other bacterial populations as well. Moreover, TPC, PSB, and NFB counts in MET-contaminated soils tended to be higher than those in LCT-contaminated soils (data not shown).

A correlation between the numbers of soil bacteria and time (t) for all treatments is presented in Table 5. It is evident that the counts of TPC, PSB, and NFB, both with and without BC/NBC application in soils contaminated with MET or LCT, followed a first-order reaction, with R^2^ values ranging between 0.8 and 1.0, except for BC5/NBC5. For BC5/NBC5, the behavior of these bacteria was well-described by a pseudo-first-order reaction, with R^2^ values between 0.9 and 1.0. Furthermore, the rate of increase in TPC, PSB, and NFB was more pronounced in soil with BC3/NBC3 application compared to BC1/NBC1. Additionally, the increase in all microbial counts tended to be higher with NBC application compared to BC.

**Table 5:** Regression results for total plate count (TPC), phosphate-solubilizing bacteria (PSB), and nitrogen-fixing bacteria (NFB) with the introduction of biochar (BC) and nano-biochar (NBC) at 1%, 3%, and 5% (w w^−1^) over time (t) in soil contaminated with methomyl (MET) and lambda-cyhalothrin (LCT).

Treatment	Methomyl (MET)		Lambda-cyhalothrin (LCT)	
	Equation	R^2^	Equation	R^2^
Total plate count				
Without BC/NBC	TPC = − 0.369(t) + 4.041	0.988	TPC = − 0.462(t) + 4.166	0.978
BC1	TPC = 0.967(t) + 2.891	0.992	TPC = 0.735(t) + 3.271	0.991
BC3	TPC = 0.992(t) + 3.064	0.993	TPC = 0.807(t) + 3.225	0.976
BC5	TPC = − 0.401(t^2^) + 2.701(t) + 1.720	0.995	TPC = − 0.336(t^2^) + 2.176(t) + 2.396	0.956
NBC1	TPC = 0.991(t) + 3.049	0.998	TPC = 0.769(t) + 3.255	0.991
NBC3	TPC = 1.029(t) + 3.055	0.992	TPC = 0.926(t) + 3.206	0.997
NBC5	TPC = − 0.391(t^2^) + 2.685(t) + 1.760	0.965	TPC = − 0.409(t^2^) + 2.691(t) + 1.716	0.978
Phosphate-solubilizing bacteria				
Without BC/NBC	PSB = − 0.387(t) + 6.489	0.927	PSB = − 0.248(t) + 5.216	0.846
BC1	PSB = 0.166(t) + 5.642	0.978	PSB = 0.385(t) + 4.369	0.985
BC3	PSB = 0.223(t) + 5.643	0.974	PSB = 0.411(t) + 4.367	0.961
BC5	PSB = − 0.078(t^2^) + 0.384(t) + 5.594	0.841	PSB = − 0.139(t^2^) + 0.993(t) + 3.818	0.867
NBC1	PSB = 0.189(t) + 5.633	0.993	PSB = 0.425(t) + 4.357	0.987
NBC3	PSB = 0.250(t) + 5.602	0.961	PSB = 0.435(t) + 4.347	0.990
NBC5	PSB = − 0.063(t^2^) + 0.321(t) + 5.652	0.899	PSB = − 0.113(t^2^) + 0.893(t) + 3.944	0.944
Nitrogen-fixing bacteria				
Without BC/NBC	NFB = − 0.267(t) + 5.281	0.977	NFB = − 0.329(t) + 4.733	0.975
BC1	NFB = 0.440(t) + 4.810	0.978	NFB = 0.333(t) + 4.263	0.986
BC3	NFB = 0.492(t) + 4.768	0.980	NFB = 0.361(t) + 4.285	0.978
BC5	NFB = − 0.229(t^2^) + 1.577(t) + 3.738	0.962	NFB = − 0.136(t^2^) + 0.892(t) + 3.882	0.966
NBC1	NFB = 0.492(t) + 4.768	0.980	NFB = 0.369(t) + 4.193	0.988
NBC3	NFB = 0.532(t) + 4.692	0.976	NFB = 0.414(t) + 4.262	0.986
NBC5	NFB = − 0.204(t^2^) + 1.370(t) + 4.022	0.965	NFB = − 0.134(t^2^) + 0.896(t) + 3.704	0.984

The impacts of BC/NBC on microbial counts involve both direct and indirect mechanisms^74^. Direct mechanisms include BC providing shelter for soil microbes through its pore structures and surfaces, supplying nutrients for microbial growth by adsorbing nutrients and ions onto its particles^77^, and potentially inducing toxicity via volatile organic compounds (VOCs) and environmentally persistent free radicals^78^. Indirect mechanisms involve BC modifying microbial habitats by improving essential soil properties such as aeration conditions, water content, and pH^77^, altering enzyme activities related to soil elemental cycles^79,80^, disrupting microbial communication via sorption and hydrolysis of signaling molecules^81,82^, and enhancing the sorption and degradation of soil contaminants, thereby reducing their bioavailability and toxicity to microbes^83–85^. However, the proposed mechanisms involved in BC/NBC-microbe interactions require further experimental verification, and research should focus on establishing the linkage between these interaction mechanisms and their environmental effects.

Regarding specific bacteria such as PSB, which are recognized for their ability to convert insoluble forms of phosphorus (e.g., iron phosphorus, calcium phosphorus, highly stable organic phosphorus, etc.) in the soil into soluble forms accessible to plants, thus enhancing nutrient availability^42,86^, studies by Ali et al.^87^, Blanco–Vargas et al.^88^, and Lu et al.^89^ support that BC acts as an effective biological inoculant, promoting the proliferation of PSB. Lu et al.^88^ also noted that functional groups on the surface of BC, such as carboxyl, amine, and amide groups, play a crucial role in facilitating the adhesion and proliferation of PSB cells. For NFB, which play a vital role in the nitrogen cycle by converting atmospheric N_2_ into forms accessible to plants through nitrogen fixation^90^, studies by Abujabhah et al.^91^, Lehmann et al.^92^, Singh et al.^93^, and Yin et al.^94^ have linked BC application to an increased abundance of N-fixing bacteria. Similar to the positive response observed in other microbial communities, the rise in NFB populations following BC/NBC application is attributed to modifications in the carbon-to-nitrogen (C/N) ratio and enhanced nutrient availability, particularly for essential elements like boron (B) and molybdenum (Mo)^95^, which are crucial for the thriving of N-fixing bacteria. Additionally, BC application improves soil physical properties, positively impacting free-living N-fixing bacteria^91^.

The observed decline in bacterial abundance following the application of BC5 in the 3rd week and NBC5 in the 2nd week aligns with findings from a meta-analysis by Li et al.^96^. A non-linear relationship between BC5/NBC5 and cultivation time suggests that several factors contribute to the negative effects of high BC/NBC application rates on microbial abundance and diversity. Excessive application can disrupt the microenvironment essential for microbial growth, impose selective pressures within microbial populations, and lead to an overall decrease in diversity^82,97,98^. Moreover, the introduction of potentially toxic components in BC/NBC may further contribute to the reduction in bacterial abundance^99^. This decline may also be linked to a decrease in SOC, as discussed earlier. Additionally, elevated C/N ratios in BC/NBC may limit carbon metabolism among microbial communities, thereby impacting their diversity^39,100^.

When examining the type of pesticides, the slight variance in bacterial counts between soil contaminated with MET and soil contaminated with LCT could stem from several factors. LCT, categorized as a hydrophobic pyrethroid insecticide, typically exhibits higher toxicity and longer persistence compared to MET, which is a hydrophilic carbamate insecticide (Table 2). Pyrethroids, known for their broad-spectrum activity and relatively high toxicity across various organisms, including soil microbes, may have a more profound effect on microbial growth^101^. Furthermore, the enduring presence of LCT in the soil prolongs microbial exposure, potentially exacerbating its impact. Conversely, while MET also exerts toxic effects on soil microbes, its shorter persistence and quicker degradation in soil^102^ might result in a comparatively lesser impact on microbial populations over time. However, it’s crucial to recognize that the precise influence of each pesticide on microbial growth within this study could vary depending on additional factors such as the adsorption capacity of BC/NBC, application rate, soil type, microbial community composition, and growing conditions. Consequently, comprehensive toxicity testing and field studies are imperative for accurately assessing the effects of pesticides on soil microbial communities with BC/NBC application.

### Influence of biochar and nano-biochar on methomyl and lambda-cyhalothrin residues in soil

**Fig. 6:**
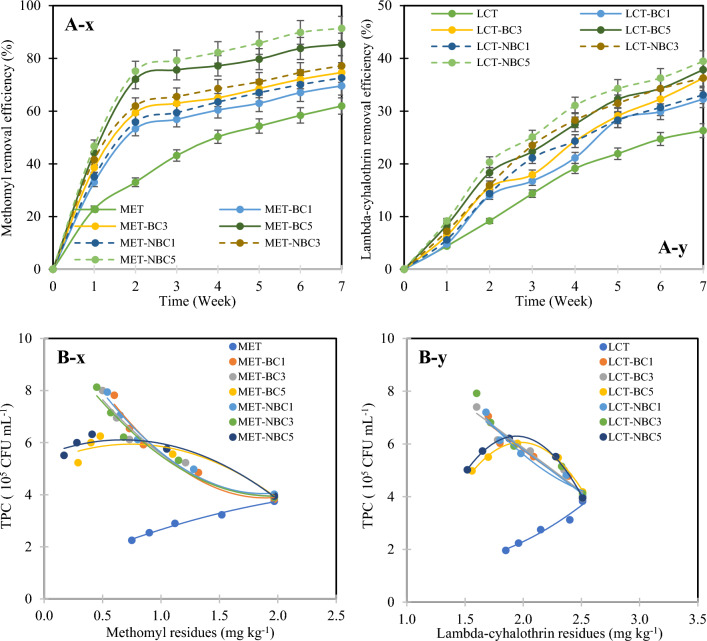
Removal efficiency of methomyl (MET) (x) and lambda-cyhalothrin (LCT) (y) with the introduction of biochar (BC) and nano-biochar (NBC) at concentrations of 1%, 3%, and 5% (w w^−1^) (**A**) and their correlations with microbial count (**B**).

Over the 7 week period, a decrease in the residues of MET and LCT was evident across all treatments (Fig. 6). In the absence of BC/NBC addition, soil samples showed a 61.9% reduction in MET residue by the 7th week (Fig. 6A-x) and a 26.3% decrease in LCT (Fig. 6A-y). Conversely, the application of BC/NBC led to a notable and significant decline (p < 0.05) in MET and LCT levels over the 7 week period. The removal efficiency of MET and LCT increased with the concentration of BC/NBC added, with a more pronounced effect observed with NBC compared to BC. Additionally, a greater reduction was noted for MET compared to LCT. Specifically, MET removal efficiency with BC5 reached 85.3%, followed by BC3 at 74.6% and BC1 at 69.5%, while NBC5 achieved an even higher efficiency of 91.4%. For LCT, the pattern was less distinct, with removal efficiencies of 37.8% for BC5, 36.3% for BC3, and 33.1% for BC1, while NBC5 resulted in a reduction of 39.4%.

The degradation kinetics of MET and LCT in soils, with and without BC/NBC application, followed a first-order equation with R^2^ values ranging between 0.9 and 1.0, as detailed in Table 6. Notably, the degradation rate of MET with BC5/NBC5 was significantly higher (p < 0.05) compared to BC1/NBC1 and BC3/NBC3. For LCT, the degradation rate with BC5/NBC5 and BC3/NBC3 showed a significant difference (p < 0.05) compared to BC1/NBC1. Likewise, the degradation rate with NBC5 application was approximately 1.3 times higher for MET and 1.1 times higher for LCT compared to BC5 application. The half-life of MET and LCT in soils without BC/NBC was 2.7 and 3.8 days, respectively. However, with BC5 application, the half-life of MET decreased to 2.1 days, and with NBC5 application, it decreased further to 1.8 days. Similarly, for LCT, the half-life decreased slightly from 3.4 days with BC5 to 3.3 days with NBC5. These findings indicate that MET degrades more rapidly in soil with BC/NBC application compared to LCT, with NBC exhibiting a slightly shorter half-life than BC at the same concentration.

**Table 6:** Regression results for methomyl and lambda-cyhalothrin reduction with the introduction of biochar (BC) and nano-biochar (NBC) at 1%, 3%, and 5% (w w^−1^) over time (t).

Treatment	Equation	t_1/2_ (day)	R^2^
Soil contaminated with MET			
MET	C_t_ = 2.025e^−0.132t^	2.72	0.961
MET-BC1	C_t_ = 1.782e^−0.151t^	2.58	0.850
MET-BC3	C_t_ = 1.722e^−0.171t^	2.46	0.851
MET-BC5	C_t_ = 1.686e^−0.245t^	2.10	0.849
MET-NBC1	C_t_ = 1.782e^−0.166t^	2.49	0.857
MET-NBC3	C_t_ = 1.695e^−0.184t^	2.39	0.858
MET-NBC5	C_t_ = 1.894e^−0.325t^	1.82	0.907
Soil contaminated with LCT			
LCT	C_t_ = 2.607e^−0.046t^	3.77	0.987
LCT-BC1	C_t_ = 2.634e^−0.058t^	3.54	0.982
LCT-BC3	C_t_ = 2.642e^−0.064t^	3.44	0.991
LCT-BC5	C_t_ = 2.593e^−0.067t^	3.40	0.976
LCT-NBC1	C_t_ = 2.601e^−0.059t^	3.52	0.972
LCT-NBC3	C_t_ = 2.601e^−0.066t^	3.41	0.969
LCT-NBC5	C_t_ = 2.571e^−0.071t^	3.34	0.959

The observed reduction in both MET and LCT content in soils, whether with or without the addition of BC/NBC, can be primarily attributed to the mode of pesticide movement in soils. This movement is largely influenced by root uptake mechanisms, as described by Miller et al.^103^, involving passive diffusion processes, as discussed by Malchi et al.^104^. Phloem transport plays a pivotal role in distributing pesticides within plant tissues, facilitating their movement from leaves to roots, fruits, and buds, as highlighted by Chen et al.^70^ and Liu et al.^105^. Additionally, the release of root exudates, such as organic acids, amino acids, and simple sugars, by plant roots can significantly contribute to the biodegradation of organic pollutants and regulate the bioavailability of redox-sensitive pollutants^36,106^. These root exudates can impact the physicochemical properties of BC/NBC, thereby influencing its capacity to adsorb pollutants. Li et al.^107^ found that oxalic acid undergoes acid oxidation when interacting with BC, increasing BC polarity and consequently enhancing its adsorption capacity. However, the diffusion of root exudates into the pores of BC/NBC could potentially disrupt the pore structure, releasing adsorbed pollutants back into the environment^108^.

Regarding the adsorption mechanisms of BC/NBC, these are governed by a combination of physical and chemical interactions. Initially, pesticides undergo physisorption, facilitated by weak intermolecular forces such as diffusion, hydrophobic interactions, π–π bonds, van der Waals forces, and hydrogen bonding^19,58,109^. FTIR analysis, as shown in Fig. 3, supports the composition of BC/NBC, indicating a high presence of unsaturated hydrocarbons, along with functional groups like ketones, carbonyls, and aromatic organic molecules. The dominant hydroxyl groups on the BC surface play a crucial role in bridging hydrogen-bonding interactions between BC and pollutants^58^. Additionally, the aromatic functional groups of BC can initiate π–π electron donor–acceptor (EDA) interactions with pollutants^60^, while the hydrophobic properties of its alkyl groups contribute to hydrophobic interactions^110^. Subsequently, chemisorption occurs through irreversible monolayer chemical interactions, such as covalent bonding or complex formation, which may act independently or synergistically^111^. The efficacy of these adsorption mechanisms depends heavily on the chemical and physical characteristics of BC, including its functional groups, mineral content, surface area, and pore structure, as well as soil properties^112,113^.

Furthermore, the introduction of BC into soil ecosystems can elicit responses from the indigenous microbial population, particularly in pesticide biodegradation. BC, especially when enriched with nutrients such as amorphous carbon and liquid organic matter, serves as a source of readily digestible microbial food, enhancing microbial activity and fostering the biodegradation of pesticides^19,109^. Supporting this, a polynomial correlation between pesticide residues, such as those of MET and LCT, and TPC was observed in this study (Figs. 6B-x, B-y), with R^2^ values exceeding 0.9 (data not shown). This observation underscores the intricate and possibly nonlinear dynamics inherent in the relationship between pesticide residues and microbial community dynamics within the soil ecosystem.

The enhanced removal efficiency of MET and LCT by NBC can be attributed to its multifaceted adsorption mechanisms, which include ion exchange, complexation, precipitation, electrostatic interactions, and physical adsorption^114^. These diverse mechanisms, whether operating independently or in combination, play crucial roles in the adsorption process of NBC^115,116^. Specifically, the presence of acidic functional groups in NBC lowers its surface potential and pH_PZC_ (Fig. 1), thereby enhancing the electrostatic effects of adsorption^117^. Electrostatic interactions have been identified as the predominant adsorption mechanism in the binding of NBC to organic pollutants^118^. Moreover, compared to bulk BC, NBC features a higher graphene structure and a larger surface area to volume ratio (as indicated in Table 2), which facilitates more frequent pore filling and π–π interactions during the adsorption process^36,119^.

The differential degradation rates observed between MET and LCT with BC/NBC can be explained by distinct mechanisms. Considering the chemical properties of MET and LCT (Table 1), LCT, being a non-polar pesticide, tends to adsorb readily onto the BC structure due to its hydrophobic nature, as influenced by the octanol–water distribution coefficient (K_OW_)^120^. Fontecha-Cámara et al.^121^ suggested that compounds with high K_OW_ values, like LCT, are primarily governed by hydrophobic and van der Waals interactions during their adsorption process. These hydrophobic interactions, driven by entropy, occur between the nonpolar alkyl side chains of LCT and the alkyl groups of BC/NBC. Additionally, the π–π electron donor–acceptor (EDA) interaction between LCT and the aromatic compounds on the BC/NBC surface, indicated by peaks at 1360–1379 cm^−1^ corresponding to aromatic C = C bonds (Fig. 3), occurs more frequently than with MET due to the higher number of aromatic rings in LCT. This π–π EDA interaction can occur between the aromatic rings of BC/NBC, which have high electron density (π −), and the electron-acceptor sites on LCT^122^.

In contrast, the presence of C = O at 1563–1587 cm^−1^ and O–H groups in the range of 1000–1500 cm^−1^ (Fig. 3) indicate that the oxygen and hydrogen in these BC/NBC functional groups participate in hydrogen bonding interactions^123,124^. In this study, MET, with its hydrophilic properties, forms stronger hydrogen bonds with BC/NBC compared to LCT. The hydrogen bonding interactions during MET adsorption likely involve the phenolic, carbonyl, and carboxyl groups of BC/NBC. These interactions could also occur between the hydrogen atoms of the carboxyl and phenolic groups of BC/NBC and the nitrogen atoms of MET^125^. These interactions facilitate the sorption of polar pesticides onto BC, utilizing hydrogen bonds that are stronger than van der Waals forces but weaker than covalent bonds^126^. Additionally, MET might be retained by BC due to its hydrophobic functional groups or could precipitate on alkaline BC surfaces^19^. MET’s smaller molecular size compared to LCT might also increase its absorption by BC, possibly due to the pore-filling mechanism^111,112^. Despite both MET and LCT being susceptible to microbial degradation^47,127^, a higher microbial population in MET-contaminated soil, potentially including species adept at MET degradation, could contribute to the greater reduction of MET compared to LCT. In summary, the combination of hydrogen bonding, pore filling, and microbial degradation mechanisms may collectively explain the observed higher reduction of MET compared to LCT by BC/NBC.

## Conclusions

The addition of BC/NBC significantly increased soil pH, and soil MC showed a noticeable rise corresponding to BC/NBC levels. SOC content declined over time in untreated soils but increased with BC/NBC addition, although a decrease was observed in BC5/NBC5 treatments after the 2nd or 3rd week. Microbial populations, including TPC, PSB, and NFB, decreased over time in MET- or LCT-contaminated soils but were significantly boosted by BC1/NBC1 and BC3/NBC3 applications. However, declines were noted in BC5/NBC5 treatments after the 2nd or 3rd week. The removal efficiency of MET and LCT increased with higher BC/NBC concentrations, with NBC exhibiting a greater effect than BC. Degradation kinetics indicated faster degradation of the hydrophilic MET compared to the hydrophobic LCT. Overall, while the BC3/NBC3 treatment appears more effective in improving soil qualities and enhancing microbial populations compared to BC5/NBC5, BC5/NBC5 outperforms other treatments, especially NBC5, in showing the highest removal efficiency and the lowest half-life for both MET and LCT. Future research should explore the long-term effects of BC, NBC, and their derivatives on pesticide behavior, soil properties, and microbial communities. Investigating potential synergies with other soil amendments could optimize soil health and pesticide mitigation. Additionally, studying the biogeochemical dynamics and environmental risks of BC and NBC is crucial. Developing technologies for NBC recovery and assessing environmental risks post-pollutant adsorption are essential future steps.

## Data Availability

All data generated or analysed during this study are included in this published article.
